# Limits to timescale dependence in erosion rates: Quantifying glacial and fluvial erosion across timescales

**DOI:** 10.1126/sciadv.adr2009

**Published:** 2024-12-20

**Authors:** Joel A. Wilner, Bailey J Nordin, Alexander Getraer, Rowan M. Gregoire, Mansa Krishna, Jiawen Li, Derek J. Pickell, Emma R. Rogers, Kalin T. McDannell, Marisa C. Palucis, Brenhin Keller

**Affiliations:** ^1^ Department of Earth Sciences, Dartmouth College, 19 Fayerweather Hill Rd, Hanover, NH 03755, USA.; ^2^ Department of Geoscience, University of Wisconsin–Madison, 1215 W Dayton St, Madison, WI 53706, USA.; ^3^ Department of Earth and Climate Sciences, Bates College, 44 Campus Ave, Lewiston, ME 04240, USA.

## Abstract

Earth’s topography and climate result from the competition between uplift and erosion, but it has been debated whether rivers or glaciers are more effective erosional agents. We present a global compilation of fluvial and glacial erosion rates alongside simple numerical experiments, which show that the “Sadler effect,” wherein geological rates show an inverse relationship with measurement timescale, comprises three distinct effects: a measurement thickness bias, a bias of erosion and redeposition, and a bias introduced by not observing quiescent intervals. Furthermore, we find that, globally, average glacial erosion rates exceed fluvial erosion rates through time by an order of magnitude, and that this difference cannot be explained by Sadlerian biases or by variations in hillslope, precipitation, or latitude. These findings support observations of increased erosion rates following Cenozoic cooling and glaciation, and reveal the importance of glacial erosion over millennial to orogenic timescales.

## Teaser

Globally, glacial erosion rates outpace fluvial, with limits to the biases introduced by measuring across different timescales.

## INTRODUCTION

The erosive nature of glaciation has been a source of debate for nearly two centuries (*1*–*4*), with glaciers and ice sheets serving as anything from a protective blanket unable to erode unconsolidated sediments (*3*) to one of the most markedly erosive agents on Earth (*2*), eroding at rates of up to 1 m per year (*5*, *6*), and even potentially limiting global mountain height by systematically outpacing tectonic uplift (*7*, *8*).

Any unifying theory of glacial erosion must contend with reported rates that vary by up to seven orders of magnitude (*5*, *9*), particularly including variation between cold-based and wet-based glaciers (*10*). Glacial erosion appears to result from not just one but several distinct physical processes, including direct abrasion (which is thought to depend primarily on basal slip and basal rock/sediment concentration) (*11*, *12*), regelation (*13*), plucking/quarrying (*14*), and “ripping” (*15*), alongside minor chemical weathering both by ice (*16*) and water (*17*). Empirically, glacial erosion rates are found to positively correlate with basal slip velocity and precipitation, though with large (multiple orders of magnitude) offsets between different glaciers (*18*).

Further complicating matters, observed sediment accumulation rates (glacial or otherwise) (*19*, *20*) and, in some cases, erosion rates (*4*, *21*, *22*) often display an inverse relationship between rate and timescale, such that longer measurement timescales yield slower rates of erosion and/or deposition—a phenomenon termed the “Sadler effect.” This relationship impedes the comparison of glacial and fluvial erosion rates (*4*), and may either imitate or mask true changes in erosion rate over time. The question of such Sadlerian effects is particularly critical in the context of ongoing debates about Cenozoic paleoclimate, wherein an increase in global and/or alpine erosion rates due to increased glaciation has been considered a potential cause or consequence of Cenozoic cooling (*23*, *24*), prompting substantial debate (*25*–*28*).

**Fig. 1.**
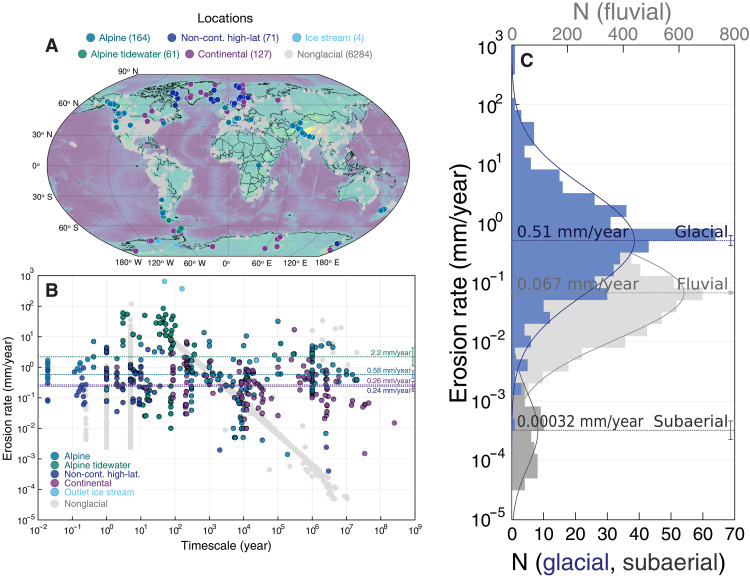
Comparison of glacial and nonglacial erosion rates. (**A**) Sample locations, categorized by glacier type. (**B**) Erosion rate as a function of timescale, on logarithmic axes, categorized by type. Log-average rates for alpine tidewater (2.2 mm/year), alpine (0.58 mm/year), continental (0.26), and noncontinental high latitude (0.24 mm/year) are shown alongside 2 SE bars. (**C**) Histogram of glacial, fluvial, and subaerial erosion rates, which are approximately normal on a logarithmic axis. Log-average rates for glacial (0.51 mm/year), fluvial (0.067 mm/year), and subaerial (0.00032 mm/year) erosion are shown alongside 2 SE bars.

To provide insight into these problems and potentially disentangle Sadlerian effects from true changes in erosion rate over time, we compile and analyze a comprehensive global dataset of over 400 glacial erosion rate measurements—roughly an order of magnitude larger than previous open compilations (*29*), spanning timescales of days to ~100 million years (Myr), across all continents and via multiple independent analytical methods. We supplement this dataset with more than 6000 nonglacial erosion rate measurements, including from preexisting compilations of basin-wide cosmogenic and volumetric erosion rates (*30*–*32*). For each glacial or nonglacial erosion rate measurement, we tabulate the method used to estimate that erosion rate (volumetric, relief-based, cosmogenic surface, cosmogenic detrital, or thermochronometric; see Materials and Methods), measurement area (km^2^), measurement timescale (years), and geographic location—through which we obtain additional parameters of interest including average annual precipitation and average regional hillslope (Fig. 1). Sample locations, as shown in Fig. 1A, span all continents and from the poles to the equator.

## RESULTS

### Systematic trends in glacial and nonglacial erosion rates

We first divide this dataset into five distinct glacier types: alpine (land-terminating glaciers hosted by tectonically active mountain ranges, but also including some non–high-latitude passive margin alpine glaciers); alpine tidewater (ocean-terminating glaciers hosted by tectonically active mountain ranges); noncontinental high latitude (glaciers neither hosted by tectonically active mountain ranges nor large enough to qualify as an ice sheet); continental (past and present continental ice sheets); and outlet ice stream (where the accumulation of a large ice sheet is channeled to a narrow active outlet glacier).

While there are visually evident differences in erosion rate between these categories, as shown in Fig. 1B (with outlet ice streams and alpine tidewater glaciers displaying the highest average erosion rates and continental and high-latitude glaciers showing the lowest), neither the dataset as a whole, nor any major glacier type appears to show a clear negative correlation between erosion rate and measurement timescale. Consequently, we suspect that any Sadlerian biases present in the dataset are not driven by the style or setting of glaciation. Moreover, as illustrated in Fig. 1C, we find that both glacial and nonglacial erosion rates approximately follow log-normal distributions, but with a characteristic log-mean erosion rate nearly an order of magnitude greater for glacial erosion (0.51 mm/year) than fluvial erosion (0.067 mm/year), and several orders of magnitude faster than nonfluvial subaerial erosion (0.00032 mm/year).

**Fig. 2.**
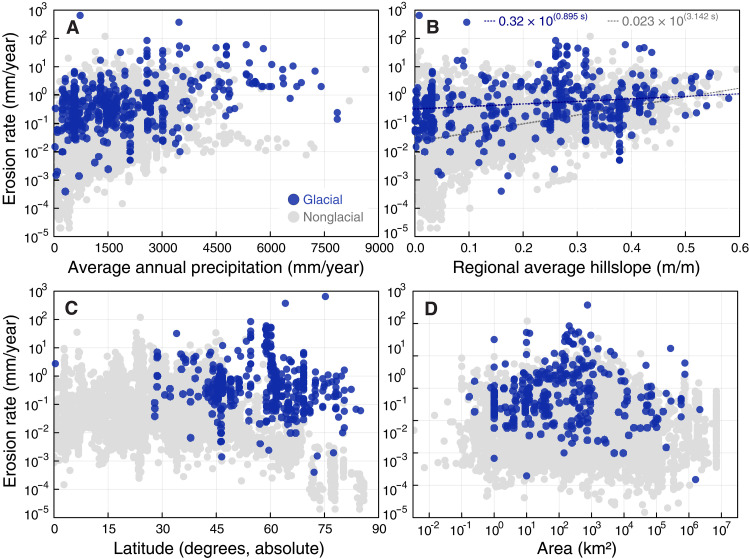
Glacial and nonglacial erosion rates as a function of potentially associated factors. These potential factors include (**A**) precipitation, (**B**) average regional hillslope, (**C**) latitude, and (**D**) measurement area.

This systematic offset between glacial and nonglacial erosion rates persists across a range of relevant independent variables (Fig. 2, A to D). Specifically, while both glacial and nonglacial erosion rates increase with precipitation (Fig. 2A) and average regional hillslope (Fig. 2B), glacial erosion rates remain systematically higher, except perhaps at very high average regional hillslopes where landsliding and mass wasting likely enhance nonglacial rates. Meanwhile, although nonglacial erosion rates generally decrease visibly toward polar latitudes (Fig. 2C), glacial erosion rates are far less sensitive, widening the gap between glacial and nonglacial erosion rates at high latitudes. Last, neither nonglacial nor glacial erosion rates appear to strongly depend on the area over which measurements are averaged (Fig. 2D).

These results differ from those of Koppes and Montgomery (*4*), who observed timescale dependence in a smaller compilation of glacial erosion rates over 10^1^ to 10^6^ year timescales. They interpreted this Sadlerian trend to reflect more rapid glacial erosion during the (warm) terminations of glacial cycles and concluded that rivers and glaciers denude landscapes at similar rates over longer timescales. The notable absence of this trend in our larger compilation suggests that (i) rapid glacial erosion is not limited to periods of deglaciation, (ii) rapid glacial erosion is constrained only in a few specific systems such as those investigated by Koppes and Montgomery (*4*), or (iii) at a global scale, this effect is masked by climatic variability.

In a sedimentary context, the Sadler effect and associated negative correlations between rate and timescale are commonly attributed to the inherently “unsteady, discontinuous” nature of sedimentation (*19*), sometimes represented quantitatively as brief pulses of deposition separated by nondepositional hiatuses drawn from a long-tailed distribution (*33*). Such mechanisms have also been proposed to apply to glacial erosion rates (*21*). However, the timescale bias expected to result from such processes is not readily apparent in our expanded glacial compilation at global scale (Fig. 1B).

**Fig. 3.**
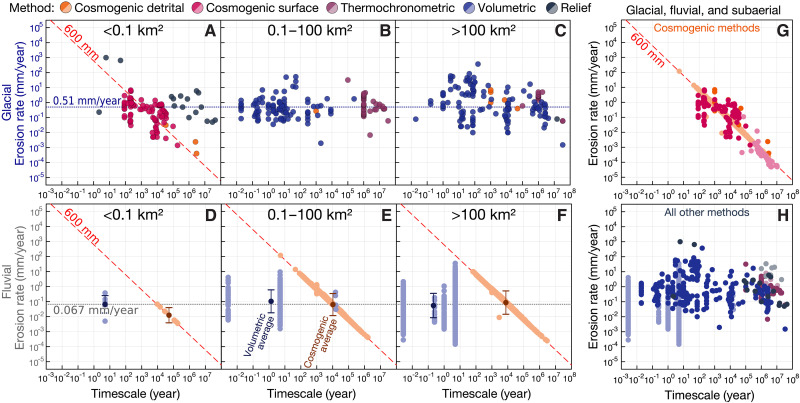
Erosion rates as a function of timescale, on logarithmic axes, categorized by measurement method and measurement area. (**A** to **C**) Glacial erosion rates versus timescale, for increasing measurement areas. Blue dotted line shows global log-mean glacial erosion rate of 0.51 mm/year. (**D** to **F**) Fluvial erosion rates versus timescale, for increasing measurement areas. Gray dotted line shows global log-mean nonglacial erosion rate of 0.067 mm/year. Error bars are 1 SD. (**G** and **H**) Combined glacial, fluvial, and subaerial erosion rates as a function of measurement timescale, contrasting cosmogenic (G) and noncosmogenic (H) methodologies. In situ cosmogenic measurements (both surface and detrital) of glacial, fluvial, and subaerial erosion scatter around a diagonal line with slope −1 representing a constant thickness of 600 mm [(A) and (D) to (G)]. Other methods appear to show less or no timescale bias (H).

In this rare event framework, we may expect that Sadlerian effects should be strongest when observing relatively small areas, while larger measurement areas may yield lower-variance measurements clustered around some true long-term mean rate that includes hiatuses (*34*). We therefore consider in Fig. 3 the influence of measurement area on timescale bias. For glacial erosion rates (Fig. 3, A to C), a Sadlerian bias does appear to emerge at the smallest areas (*<*0.1 km^2^), but this bias is confined to measurements made by in situ cosmogenic methods. For fluvial erosion rates, a very clear log-linear rate-timescale correlation emerges for detrital cosmogenic measurements regardless of basin area (Fig. 3, D to F). In both cases, this trend clusters around a thickness of 600 mm and is observed across cosmogenic measurements in our dataset (Fig. 3G). As previously noted (*34*), this trend is characteristic of in situ cosmogenic erosion rate measurements, reflecting the average penetration depth of cosmogenic rays into crustal rocks, and as such represents a measurement thickness bias.

Despite this apparently inherent limitation of surface and detrital in situ cosmogenic erosion rates, we note that for fluvial erosion (Fig. 3, D to F), global log-mean erosion rates are consistent between cosmogenic (orange error bars) and volumetric (blue error bars) methods across all but the smallest area bin. Consequently, we consider that it may be possible to partly “see past” measurement thickness biases by comparing technique-wide representative averages as long as both techniques do not share the same measurement thickness bias. We further note that these averages agree with each other and with the overall global log-mean fluvial erosion rate of 0.067 mm/year, despite differing in timescale by four orders of magnitude—suggesting the existence of a true long-term log-mean erosion rate, and implying that timescale dependence may not be an inherent physical property of erosional systems.

## DISCUSSION

### The ternary Sadler effect

Clearly, more than one process can produce negative trends in rate-timescale space; the measurement thickness bias evident in cosmogenic erosion rate determinations is notably distinct from the physical “unsteadiness” hypothesized to drive the Sadler effect more broadly. Here, we hypothesize that what has frequently been called the Sadler effect is not one singular phenomenon, but three: the erosion-redeposition effect, the nonobservation effect, and the measurement thickness effect.

Measurement thickness bias, as described above, is perhaps the simplest of the three: If a given technique measures the time it takes to erode or deposit a specific thickness of material, all rates measured with that technique will necessarily scale inversely with timescale, with a slope of −1 in log rate-timescale space. While such bias does not render measurements incorrect or inaccurate, it does impose constraints on the statistically valid use thereof. In our compilation, only cosmogenic measurements appear to strongly display this bias (Fig. 3G), but weaker biases may be theoretically expected for any technique that measures the time it takes to erode or deposit a limited range of thicknesses—including, for example, field-measured sedimentary bed thicknesses (biased toward approximately human scale) and single closure-temperature thermochronology (biased toward the depth of closure in a geothermal gradient). This bias is not inherent to the physical nature of a geologic system or process being studied, only a limitation of our ability to measure that process.

In contrast, the other two components of the Sadler effect both involve the “unsteady, discontinuous” nature of sedimentary processes originally noted by Sadler (*19*). However, we believe that it is important to separate two fundamentally distinct components of that unsteadiness: the bidirectionality of geologic processes, such that previous progress may be undone (here termed the erosion-redeposition effect), and the temporal discontinuity of geologic processes, with brief events separated by long periods of quiescence (potentially resulting in the nonobservation effect). In a sedimentary context, these two processes are easily conflated, as both lead to sedimentary hiatuses—unconformities—but the former are erosional while the latter are nondepositional. However, this distinction is critical in the context of erosion rates, because while erosional processes may be temporally discontinuous, at many scales, they are critically unidirectional. For example, sediment eroded from a fluvial catchment will not naturally return upstream against gravity. Likewise, the glacial till in a terminal moraine will not be carried uphill to its source and placed again underneath the glacier. Such unidirectionality may extend to spatial scales as small as a single ventifact under aeolian erosion, or as large as the exposed continental crust as a whole—but in either case stands in stark contrast to the bidirectional erosion-redeposition processes ubiquitous below base level in depositional environments, such as local sediment reworking in tidal or deltaic settings. In addition, nonglaciated mountain ranges typically store small volumes of sediment, so hiatuses in sedimentary records may reflect predominantly nondepositional gaps. In contrast, glaciated mountain ranges can store large volumes of sediment in overdeepened valleys where erosion and redeposition are more active, making it harder to distinguish between erosional and nondepositional hiatuses.

**Fig. 4.**
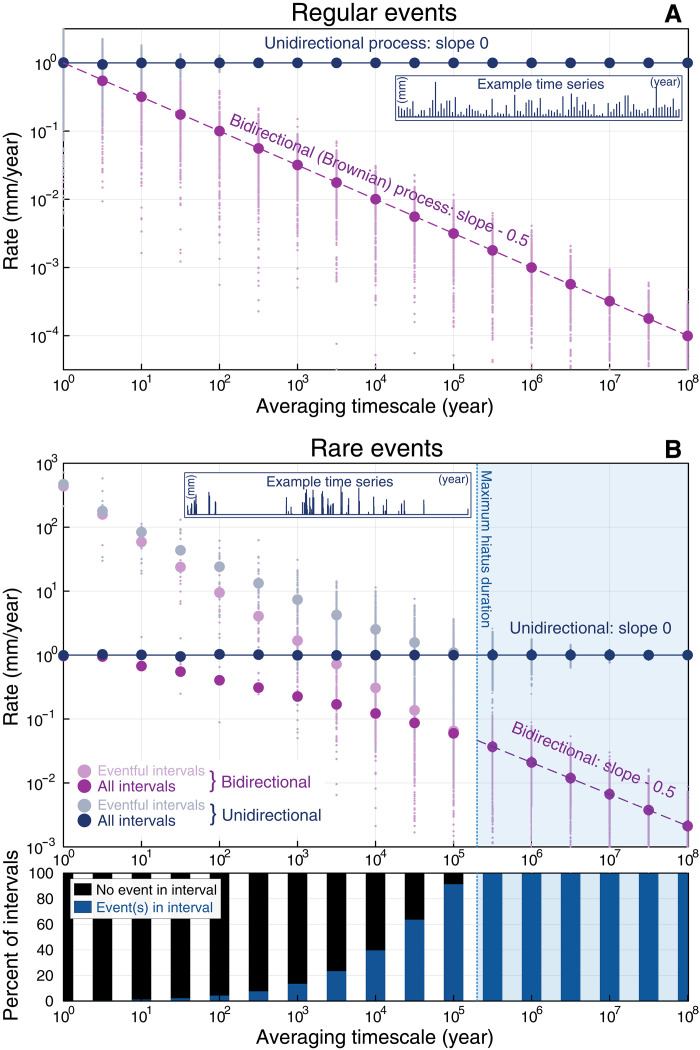
Numerical model results for regular and rare erosional and depositional events. (**A**) Unidirectional and bidirectional regular processes, where a random amount of erosion (unidirectional, blue) or erosion and deposition (bidirectional, purple) occurs regularly every year. Means of all 100,000 simulations are shown as larger blue and purple circles, while smaller dots illustrate 500 representative individual simulations. Inset: an example unidirectional time series with 100 events. (**B**) Unidirectional and bidirectional rare event processes, where events are separated by intervals of time drawn from a truncated Pareto distribution. In contrast to a regular process, here the probability of observing an erosional or depositional event depends on measurement interval duration; many short intervals contain no events (bottom). We consequently consider both mean rates when such uneventful intervals are not or cannot be observed (light blue, light purple), and mean rates when all intervals can be observed without bias (dark blue, dark purple). Inset: an example unidirectional time series with 100 events.

To better understand the effects of bidirectionality and temporal discontinuity in rate-timescale space, we conduct a number of numerical experiments. First, we consider regular events—that is, events proceeding at fixed intervals in time. As further discussed in Materials and Methods, we simulate regular events with a frequency of 1/year and a mean absolute value of 1 mm, either all positive (a half-normal distribution; unidirectional) or equally positive and negative (a symmetrical normal distribution; bidirectional). As shown in Fig. 4A, when averaged over 100,000 iterations, the unidirectional process usually has a constant mean rate of 1 mm/year across all timescales. The bidirectional process, which is effectively equivalent to one-dimensional Brownian motion, has no well-defined long-term rate but rather a slope of −0.5 in log-rate versus log-timescale space. This is a consequence of the well-known result (*35*) that the expected absolute displacement of a Brownian particle over time *t* is proportional to $\sqrt{t}$, so the expected rate scales as $\sqrt{t}/t$, or *t*^*−*0.5^. Consequently, for regular processes, we may only expect geologic rates to be inherently timescale dependent in the case of bidirectional processes (that is, the erosion-redeposition effect).

We next consider irregular events, where the time between erosional or depositional events follows a long-tailed rare-event distribution. Specifically, we follow Ganti *et al.* (*21*) in drawing hiatus durations from a truncated Pareto distribution with a maximal value of 200,000 and a shape parameter of 0.5, while event magnitudes are again half-normal (unidirectional) or normal (bidirectional), as further discussed in Materials and Methods. As noted by Ganti *et al.* (*21*), to avoid introducing a bias that the observed interval always begins with an event, we must, for each simulation, first construct a time series of events longer than both the maximum hiatus duration and the longest simulated averaging timescale. We then choose the start of our averaging interval randomly within this preconstructed time series.

The proportion of intervals that contain an erosional or depositional event, again over 100,000 simulations, is shown at the bottom of Fig. 4B. At shorter averaging timescales, most intervals contain no erosional or depositional events. The proportion of such “uneventful” intervals decreases to zero at timescales longer than the maximum hiatus duration, whereafter both processes behave as they would in the regular event model above—unidirectional processes show no timescale dependence, while bidirectional (erosion-redeposition) processes display a slope of −0.5 (Fig. 4B). At timescales shorter than the maximum hiatus duration, mean rates diverge depending on whether such hiatus-only uneventful intervals are included in the tabulation. This distinction is notable given that such intervals may be unlikely to be studied (an observational bias), or potentially impossible to measure by some techniques (a measurement bias). As shown in Fig. 4B left of the maximum hiatus duration, if only “eventful” intervals are included in the mean, even unidirectional processes show a distinct timescale dependence (again with a slope of approximately −0.5), as in the results of Ganti *et al.* (*21*), and bidirectional processes are steepened to slopes more negative than their otherwise characteristic −0.5. However, if the set of intervals included in the mean is not biased against uneventful intervals, unidirectional processes once again show no discernible timescale dependence. Given that this form of Sadlerian bias arises when zero-rate intervals are not observed, we term it the nonobservation effect.

### Implications for Cenozoic climate and erosion

Our results reveal that long-term log-mean glacial erosion rates exceed fluvial rates by approximately an order of magnitude and that this discrepancy cannot be explained by Sadlerian biases or by variations in precipitation, latitude, or average regional hillslope. To first order, our results thus appear to be consistent with the proposal that observed cooling and glaciation in the Cenozoic could have enhanced global high-latitude and/or alpine erosion rates (*23*, *24*, *36*, *37*). This proposal has been the subject of much discourse (*25*, *26*, *28*, *38*) and plays a central role in the broader debate about the interplay between tectonics, climate, and erosion, the Cenozoic seawater Sr-isotope record, and the ultimate cause of Cenozoic cooling. The Cenozoic seawater Sr-isotope record has long been interpreted to represent increased erosion as a result of glaciation (*36*), tectonics (*39*), or a positive feedback between the two (*40*, *41*), though some work has also suggested that the high ^87^Sr/^86^Sr ratio might result from reduced weathering of the Himalaya (*42*).

Objections to such an increase in erosion rate were originally raised on the basis that an unbalanced increase in silicate weathering could rapidly consume all the CO_2_ in the atmosphere (*43*). However, recent studies of sulfide and organic carbon weathering suggest that the total CO_2_ response to increased erosion is much more closely balanced (and more lithology dependent) than previously expected (*44*, *45*), especially in glacial environments, where weathering may even act as a net source of CO_2_ (*46*). Instead, the most notable direct counterevidence at present to the proposal of increased Neogene erosion is the stability of oceanic^10^Be/^9^Be and ^7^Li/^6^Li ratios.

The ratio of ^10^Be/^9^Be, in contrast to in situ cosmogenic Be, in principle represents meteoric deposition of atmospherically produced cosmogenic ^10^Be relative to erosional supply of ^9^Be. As such, it is not subject to the inherent measurement thickness bias that is unavoidable in in situ measurements. Measurements of deep-sea chemical sediments such as seafloor Fe-Mn crusts suggest that oceanic ^10^Be/^9^Be has been approximately stable since *>*10 million years ago (Ma) globally across multiple ocean basins (*25*), suggesting apparently stable rates of weathering through the Cenozoic.

Meanwhile, seawater ^7^Li/^6^Li, which has been interpreted to record weathering fluxes due to the fractionation of Li isotopes during chemical weathering and reverse weathering, has been stable since *>*8 Ma (*38*, *47*). Together, these observations have been interpreted to suggest that Cenozoic atmospheric CO_2_ drawdown may primarily reflect increased land-surface reactivity (*48*). In this context, one of the simplest ways to increase the chemical reactivity of Earth’s continental crust is to increase the surface area exposed to weathering processes by reducing grain size, that is, comminution—a process frequently associated with glacial erosion.

In addition to promoting increased reactivity through comminution, glacial erosion may act in several ways to mask the impact of increased erosion on ocean ^7^Li and ^10^Be. Regarding the ^7^Li/^6^Li proxy, glacial rivers have been observed to be less enriched in ^7^Li than nonglacial rivers (*49*), potentially in part due to the incorporation of highly comminuted rock flour, which is unfractionated relative to bedrock—consistent with the observation of a low δ^7^Li colloidal end member by Wimpenny *et al.* (*49*). Consequently, increasing the proportion of glacial erosion may allow for increased erosion and weathering at constant seawater δ^7^Li.

**Fig. 5.**
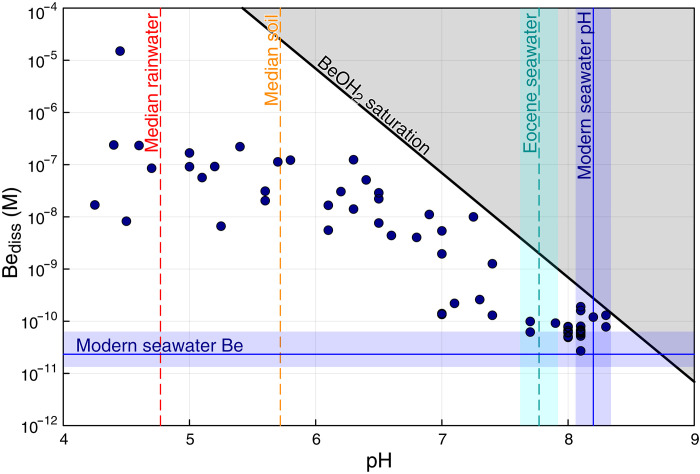
Dissolved beryllium concentrations as a function of pH for terrestrial surface waters. pH values are compiled from the literature (table S3), alongside the BeOH_2_ saturation line corresponding to a *K*_sp_ of 10^*–*21*.*16^ (*96*). In general, dissolved beryllium concentration Be_diss_ consistently decreases with increasing pH. For comparison, modern seawater Be (*97*), modern seawater pH (*51*), Eocene seawater pH (*98*), median rainwater pH (*50*), and soil water pH (*50*) are also shown.

The impact of glacial erosion on seawater ^10^Be/^9^Be ratios is dependent on the balance between cosmogenic ^10^Be deposition and the supply of eroded ^9^Be (*25*), with observations suggesting that only about 2% of eroded ^9^Be is supplied to the ocean in dissoluble form (Supplementary Materials). This ratio is sensitive to pH levels, with higher oceanic pH potentially reducing the solubility of ^9^Be (*50*, *51*)—as shown in Fig. 5 as a decline in dissolved Be concentrations by about three to five orders of magnitude between pH 4 and 8. Because glacial erosion produces finely comminuted but relatively unweathered material, any subsequent chemical weathering at oceanic pH conditions may further limit ^9^Be’s mobility (*52*), complicating interpretations of ^10^Be/^9^Be as a proxy for past erosion rates. These considerations regarding the impact of glacial erosion on the ^7^Li and ^10^Be cycles, together with a modern understanding of coupled silicate, organic carbon, and sulfide weathering (*44*, *45*), suggest that the basic hypothesis of increased Neogene erosion as proposed by Raymo *et al.* (*39*) and others may be broadly correct, especially if substantially mediated by glacial erosion.

### Broader implications for global glacial erosion

We find that the Sadler effect comprises three distinct hitherto undescribed phenomena: the erosion-redeposition effect, resulting from the bidirectionality of sedimentary processes; the nonobservation effect, resulting from the intersection of long-tailed hiatus duration distributions with observational biases against hiatuses; and the measurement thickness effect, an artifact of techniques that measure the time taken to erode or deposit a specific thickness of material. Previously, the Sadler effect has been taken to imply that there is no well-defined long-term rate in a system with Sadlerian timescale-rate scaling. However, our results suggest that this is only true if the erosion-redeposition effect applies, as it is the only effect resulting from physical processes as opposed to measurement limitations. Our numerical experiments demonstrate that for unidirectional processes, long-tailed hiatus duration distributions are insufficient to produce Sadlerian effects when the time intervals included in the mean form a representative sample set (i.e., not biased against hiatus-only intervals), thus avoiding the nonobservation effect. We further show that while certain measurement techniques (particularly in situ cosmogenic nuclide measurements) do have inherent timescale biases, these biases are not insurmountable and do not prohibit the determination of true timescale-independent average erosion rates.

After accounting for Sadlerian effects, we show using our comprehensive global dataset that average glacial erosion rates are an order of magnitude faster than average fluvial erosion rates. This disparity persists across a range of relevant independent variables, including precipitation, average regional hillslope, latitude, and measurement area, and becomes more pronounced toward polar latitudes—and across timescales *<*1 year to *>*10^6^ years, suggesting that rapid glacial erosion is not constrained to periods of deglaciation. Our results further suggest a negative feedback on elevation where mountains tall enough to become glaciated are eroded more rapidly, consistent with the evidence for a “glacial buzzsaw” effect (*7*, *8*). These results are reassuring given that the reverse (slower glacial than fluvial erosion) would instead lead to a bimodality-inducing positive feedback where tall mountains get taller and short mountains get shorter, contrary to observed global elevation distributions.

It has not escaped our notice that observed glacial erosion rates, even based on the slower rates characteristic of Cenozoic continental ice sheets (~0.26 mm/year), would be sufficient to produce the hypothesized 3 to 5 km of erosion associated with the Great Unconformity (*53*, *54*) in ~12 to 19 Myr—about a quarter of the duration of known Neoproterozoic glaciation. That said, even the slower rates characteristic of fluvial erosion (~0.067 mm/year) would suffice over a somewhat longer interval; we thus suggest that the limiting factor in the development of such unconformities is likely global base level, not absolute erosion rate. In this context, the most important difference between fluvial and glacial erosion is instead the persistently lower erosional base level associated with extensive ice sheets. Nonetheless, greater glacial than fluvial erosion rates are highly notable in a broader climatic and tectonic context. Our results thus far support the hypothesis that Cenozoic cooling may have enhanced erosion rates at higher latitudes and elevations (*37*, *40*). Future work may consider the distribution of glacial erosion rates presented here as a statistical constraint on erosion parameterizations in ice sheet models—coupled with ice dynamics, such parameterizations would improve our quantitative understanding of spatiotemporal variations in Cenozoic glacial erosion as well as the erosive potential of modern and ancient ice sheets.

## MATERIALS AND METHODS

### Observed erosion rates

Glacial and nonglacial erosion leave characteristic signatures on both the material they erode and the landscapes left behind. Common methods used to reconstruct these erosion rates, and those compiled in this study, include sediment volumetric or yield data, relief, cosmogenic radionuclide-derived rates (surface, detrital), and low-temperature thermochronometry-derived rates. Different techniques measure across different time scales of integration (i.e., daily up to million-year time scales) as well as different spatial scales (e.g., low-temperature thermochronometry are point measurements while sediment yield measurements are inherently spatially averaged over the catchment of interest). We briefly describe each technique below.

### Sediment volume and yield data

The volume of sediment that has accumulated within a deposit has commonly been interpreted as a measure of the erosive history of its upslope catchment (*23*, *55*). This approach is applied by dating geomorphic landforms, sedimentary outcrops, oceanic drill cores (*56*–*64*), and, more recently, from seismic differencing techniques (*5*, *65*, *66*). Sediment yield is primarily the suspended sediment load in rivers, dominated by clay- to silt-sized particles, and provides an estimate of the rate of mechanical denudation within a basin. The errors on erosion rates from sediment volume measurements will depend on the precision of mapping of individual stratigraphic or geomorphic units, and physically controlled spatial variability of sedimentation. Errors from sediment yield data can result from storage in the catchment and inadequate data sampling intervals.

### Relief

Another metric for estimating erosion rates involves averaging observed differences in surface relief over the time span during which that material was removed. Some of the earliest estimates of glacial erosion (*56*, *67*) hinge on exposures of preglacial landscapes as evidence of large-scale glacial erosion. Isostatic rebound and uplift as a response to extensive glacial erosion have also been used to estimate past erosion (*68*–*70*). More recent studies have been able to reconstruct erosion through differences in surface elevation of ice or differences in bedrock elevation from seismic surveys (*5*, *71*). These estimates involve substantial assumptions about timescales of relief generation and oversimplification of erosive processes. However, fundamentally, erosion rate estimates from differences in relief are calculated from measured thicknesses and, thus, do not incorporate the same systematic error as other methods.

### Cosmogenic radionuclides

To assess erosion rates over 10^2^ to 10^5^ years, we used data from in situ produced terrestrial cosmogenic radionuclides. Cosmogenic nuclides like ^10^Be and ^26^Al are produced by cosmic radiation at a rate independent of erosion rate (*72*–*74*), but can only constrain exhumation to a depth of 0.6 m, below which cosmogenic rays do not attenuate (*75*).

Because cosmogenic nuclides are produced only in rocks exposed at the surface and not covered by ice, the depth of subglacial erosion and the duration of ice cover can affect the amount of cosmogenic nuclides that are present in bedrock (*73*, *74*, *76*, *77*). Surface cosmogenic nuclide concentrations therefore contain information about both the extent of erosion and the burial history at a given location. Because erosion rates calculated from cosmogenic nuclides in bedrock samples require inferring a spatially averaged rate from a single-point measurement, there is potential to obscure true temporal and spatial variability.

Erosion rates deduced from detrital cosmogenic nuclide concentrations, typically from river sediments, represent spatially averaged rates. Detrital-based erosion rates reduce some of the complications of single-point measurements, but still involve spatial and temporal averaging. In addition, it should be noted that cosmogenic nuclides like ^10^Be are abundant in deeply weathered material like soil and till due to prolonged exposure at the surface but absent in freshly exposed bedrock (*9*, *78*). High nuclide concentrations in detrital settings like tills, moraines, riverbeds, and ocean sediments therefore indicate a remobilization of surface sediments rather than exposure of fresh bedrock (*79*–*82*).

### Low-temperature thermochronometry

To assess erosion rates on million-year time scales, low-temperature thermochronometric methods such as apatite and zircon (U-Th)/He (AHe and ZHe, respectively), apatite and zircon fission track (AFT and ZFT, respectively), and ^4^He/^3^He thermochronometry are used (*83*). The most simplistic approach for estimating exhumation magnitude (and rates) is by determining how much time has passed since a given rock parcel has transitioned through its apparent isotopic closure temperature (*84*). The accrued time since closure is the effective “cooling age” and the temperature sensitivity of the thermochronometer is related to depth (i.e., elevation) assuming some reasonable paleo-geothermal gradient (*85*, *86*). Collectively, the aforementioned methods span sensitivities of *>*200*°*C to ~30*°*C, or upper crustal depths of ~0.4 km to *>*7 km assuming a surface temperature of 20*°*C and an average geothermal gradient of 25*°*C/km. In glacial environments, coupled “age-elevation” exhumational information can yield the duration and intensity of glacial erosion (*87*). These low-temperature thermochronometers constrain long-term (10^4^ to 10^7^ years) exhumational histories in line with timescales over which glaciers typically erode. Detrital tracer thermochronology is also a useful tool to understand glacial catchment erosion (*88*, *89*). The analysis of detrital minerals provides a spatially integrated cooling signal of the total catchment where sediment is sourced and can also provide information on erosion from inaccessible locations under glaciers (*90*).

### Global compilation

The data were manually extracted from the published literature, and we compiled a full reference list that can be found along with all other code and data in our online repository. These data span the globe (Fig. 1A) and cover regions of glaciation from the present to the late Neoproterozoic, with the areas of the measurements ranging from single-point localities (10^*−*8^ km^2^) to continental (10^6^ km^2^), and temporally span from ~600 Ma (*91*) to the present. The time intervals of the data points encompass the orders of magnitude between months to hundred millions of years. The glaciers in the dataset are categorized into alpine (land-terminating glaciers typically hosted by active plate boundaries, but also including some non–high-latitude passive margin alpine glaciers), alpine tidewater (ocean-terminating glaciers hosted by tectonically active mountain ranges), continental, noncontinental high latitude (not active plate boundary), and outlet ice stream. We specifically exclude rates that reflect periglacial processes (e.g., environments that were previously glaciated but are now weathering by subaerial processes).

We compiled nonglacial erosion rate data in the same manner. A full reference list is included in our online repository. Similar to glacial erosion rates, our nonglacial data span the globe (Fig. 1A) and are broadly representative of time intervals and measurement areas. We include both fluvial and subaerial (including periglacial) erosion in our compilation, although the majority of the data (*>*96%) is fluvial.

Average annual precipitation was estimated for each terrestrial sample locality using the CHELSA climatology (*92*). Average regional hillslope at each terrestrial sample locality was calculated using the Shuttle Radar Topography Mission at 15 arc sec resolution (SRTM15+) Digital Elevation Model (DEM) (*93*) over a 18 *×* 18 km moving window (the median size of all basins in our analysis). The hillslope represents the average of all grid point–wise slopes in the basin, where the grid point–wise slope is taken to be the greatest of the eight possible diagonal slopes from a point to its eight nearest neighbors in a rectangular grid (the D8 flow direction).

To test the impact that bedrock lithology may have on erosion rate, we assigned each sample to a sedimentary, igneous, or metamorphic lithology using the generalized geologic map of the world of (*94*), accessed using the Macrostrat API (https://macrostrat.org/burwell) (*95*). When possible, metamorphic rocks are categorized as their protolith (e.g., metaigneous rocks are considered igneous). While the resolution of the map is very coarse, this general geologic map was chosen as sample locations are often uncertain, and the areas of erosion rate measurements are often very large. We were able to retrieve bedrock lithologies for ~90% of our glacial sites and ~90% of our nonglacial sites. While bedrock lithology under continental ice sheets is unknown, most erosion rates are taken from the edge of ice sheets, where lithology is better constrained. The lithology of continental ice sheets is therefore not necessarily representative of the eroded lithology.

### Statistical analysis

We performed analysis of covariance (ANCOVA) tests to determine whether the Sadlerian trends (if apparent) of the different methodologies differ significantly from one another. ANCOVA is a statistical method that combines aspects of analysis of variance (ANOVA) with regression analysis, allowing for the examination of the influence of a continuous independent variable (here, timescale) on a continuous dependent variable (here, erosion rate) while controlling for the impact of categorical covariates. The pairwise comparison ANCOVA results indicate that Sadlerian slopes of the cosmogenic methods (detrital and surface) differ significantly from the other three methods when considering glacial plus nonglacial samples together for all pairs except cosmogenic surface and thermochronometric (table S1). The Sadlerian slope associated with the volumetric method is significantly shallower than that of all other methods for both the glacial-only and glacial plus nonglacial cases, with the exception of relief versus volumetric for glacial plus nonglacial. We further delineate between methodology and glacier type to visualize Sadlerian slopes for these parameters when taken in combination (fig. S3). We find that the most profound Sadlerian trends occur for nonglacial cosmogenic samples (detrital or surface) and for alpine glacier cosmogenic surface samples. Taking nonglacial and glacial samples into account together, cosmogenically derived Sadlerian slopes map as approximately −1, consistent with our prior discussion. Volumetrically derived samples have a near-zero Sadlerian slope regardless of glacier type.

## Supplementary Materials

### The PDF file includes:

Supplementary Notes Figs. S1 to S4 Table S1 Legends for data S1 and S2
