# Supplementary material for: Limits to timescale dependence in erosion rates: Quantifying glacial and fluvial erosion across timescales
Source: Sci Adv. 2024 Dec 20;10(51):eadr2009. doi: 10.1126/sciadv.adr2009 (PMC11661439; doi:10.1126/sciadv.adr2009)
Supplement: Supplementary file 1 — Supplementary Notes Figs. S1 to S4 Table S1 Legends for data S1 and S2 [file sciadv.adr2009_sm.pdf]

Science Advances
AAAS

Supplementary Materials for

Limits to timescale dependence in erosion rates: Quantifying glacial and fluvial erosion across timescales

Joel A. Wilner *et al.*

Corresponding author: Joel A. Wilner, joel.a.wilner.gr@dartmouth.edu;
Bailey J. Nordin, bailey.j.nordin.gr@dartmouth.edu; Marisa C. Palucis, marisa.c.palucis@dartmouth.edu;
C. Brenhin Keller, cbkeller@dartmouth.edu

Sci. Adv. **10**, eadr2009 (2024)
DOI: 10.1126/sciadv.adr2009

The PDF file includes:

Supplementary Notes
Figs. S1 to S4
Table S1
Legends for data S1 and S2

Other Supplementary Material for this manuscript includes the following:

Data S1 and S2

P9. Figure S3

P10. Figure S4

P11. Table S1

Supplementary Materials

Numerical experiments

Regular processes

We simulate regular events by drawing for each model year a pseudorandom distance from a Normal distribution with a mean of 0 mm and standard deviation of $\sqrt{\pi/2}$ mm, such that the mean absolute deviation of the distribution is 1 mm. For a bidirectional process, positive and negative numbers represent the two competing directions of the process (e.g., erosion and deposition) in one dimension. For a unidirectional process, we take instead the absolute value of this number. We repeat this procedure 100,000 times for each timescale and tabulate mean rates.

Rare event processes

We follow (*21*) in drawing hiatus durations (in years) from a truncated Pareto distribution with a maximal value of 200,000 and a shape parameter of 0.5. Event magnitudes are again drawn from a Normal distribution, though now one with a mean absolute deviation (in millimeters) equal to the mean of the truncated Pareto distribution (in years) to retain approximately unit rate scale in mm/yr. While a Pareto distribution with shape parameter less than one has an infinite mean, the bounded (or truncated) Pareto has a finite mean regardless of shape parameter. A truncated Pareto with shape parameter 0.5 and maximal value 200,000 has a mean of about 447.21.

As noted by (*21*), to avoid introducing a bias that the observed interval always begins with an event (or more subtly, always begins at the time we begin observing it – as this will not

be the case in the natural world), we must first construct a timeseries of events longer than both the maximum hiatus duration and the longest averaging timescale we wish to simulate (in our case, 10^8 years). We then choose the start of our averaging interval randomly within this preconstructed timeseries. We repeat this procedure 100,000 times and tabulate mean rates for each averaging timescale.

To reproduce the numerical model of (*21*), we generated random time series of low erosion hiatuses separated by pulses of rapid erosion. Hiatus lengths (x [y]) were sampled from a truncated Pareto distribution, described in Equation S1.

$$x = \left(1 - U\left(1 - H^{-\alpha}\right)\right)^{-\frac{1}{\alpha}}. \tag{S1}$$

where U is random and uniformly distributed on $(0, 1)$, H [y] is the maximal hiatus length, and α is the tail-index. Following (*21*), we used $\alpha = 0.5$ and $H = 200$ ky to generate a series of hiatuses and excluded the first 500 ky to ensure that inferred erosion rates were independent of initial model conditions. We rounded the values so that hiatuses had units and resolution of one year, and took the cumulative sum to create a time series of erosion events.

From Eq. S3, we calculated erosion rates (E_{obs}) as follows in Eq. S2.

$$E_{obs}(T) = E_{hiatus} + \frac{m}{T}\sum_{i=1}^{n} Y_i \tag{S2}$$

where T [y] is the averaging time-scale or time before present, Y is the series of erosion events younger than T, m [mm] is the magnitude of erosion events, and E_{hiatus} [mm/y] is the baseline erosion rate during hiatuses. (*21*) used $m = 10$ mm and $E_{hiatus} = 0$. Here we use the same value for m but chose $E_{hiatus} = 0.0002$ mm/y to reflect a landscape typified by slow and steady erosion punctuated by brief pulses of rapid erosion.

The timescale dependence of estimated erosion rates is inherently dependent on the recurrence probability of erosional pulses. (*21*). For any timescale T, the estimated erosion rate E_{obs}

can be expressed as an average of the magnitude of erosional pulses Y_i sampled during that timescale (described in Eq. S3) (*21*).

$$E_{obs}(T) = \frac{1}{T}\sum_{i=1}^{n} Y_i \tag{S3}$$

Heavy-tailed distributions are typically not exponentially bounded, with a cumulative distribution function of the form $t^{-\alpha}$. This type of distribution will have an infinite mean if $\alpha < 1$. As such, per the law of large numbers, if these hiatuses were to have a heavy-tailed distribution with an infinite mean value, then E_{obs} need not converge (*21, 33*). When erosion rates obey a heavy-tailed distribution (such that $\alpha < 1$), then estimated erosion rates will display an inverse power-law dependence on T (for the detailed derivation see (*33*)). Two caveats may apply; first, infinite duration hiatuses are not possible in real-world samples, therefore all real-world heavy-tailed distributions are truncated. Second, this inverse power-law dependence only emerges when intervals are constrained to start or end with an event, or equivalently, when zero rate "uneventful" intervals are excluded.

Beryllium isotope mass balance considerations

To better understand the impact of glacial erosion on the seawater ^{10}Be/^{9}Be proxy, we must consider the mass balance of cosmogenic and eroded beryllium. The average meteoric ^{10}Be flux has been estimated at 1.23×10^6 atoms/cm^2/yr (*96*), corresponding to 6.27×10^{24} atoms/yr ^{10}Be globally. In comparison, rivers supply $\sim$19.1 Pg suspended and 3.8 Pg dissolved solids to the ocean annually (*97*) as a result of weathering and erosion of the continental crust. Since the continental crust generally averages $\sim$2.1 PPM Be (*98*) we may estimate this flux to supply a total of 4.81×10^{10} g/yr $\approx 3.21 * 10^{33}$ atoms/yr ^{9}Be globally. To first order, excluding minor additional fluxes of *in situ* ^{10}Be and hydrothermal ^{9}Be (*96*), we may then expect a modern ocean ^{10}Be/^{9}Be ratio of approximately 1.9×10^{-9}. Observed ocean ^{10}Be/^{9}Be ratios are on the order

of 1×10^{-7} (*25*) — nearly two orders of magnitude higher. This discrepancy suggests that only ~2 percent of eroded ^{9}Be is supplied to the ocean in dissoluble form, in comparison with most or all cosmogenic ^{10}Be. Though this discrepancy may be explained by the dispersed atomic production and meteoric deposition of ^{10}Be in contrast to the mineral form of eroded ^{9}Be, the accuracy of ocean ^{10}Be/^{9}Be as a proxy for the erosion of the continental crust then depends critically on the constancy of the fraction of eroded ^{9}Be that reaches the ocean in dissoluble form.

As a result of this discrepancy, one interpretation may be to consider the ^{10}Be/^{9}Be proxy to be more accurately a weathering proxy than an erosion proxy. Such an interpretation would substantially diminish the conflict between ^{10}Be/^{9}Be stability and potential variation in global erosion rates over the past 10 Myr. If we consider instead only the 3.8 Pg/yr dissolved flux as an estimate of the mass of continental crust subject to chemical weathering each year (possibly a conservative one, as clays produced by incongruent weathering will contribute to the suspended flux rather than the dissolved flux), and again assume this weathered crust contains ~2.1 PPM ^{9}Be (again perhaps an underestimate, as weatherable feldspars contain more Be than unweatherable quartz), then we obtain a conservative estimate of 7.98×10^{9} g/yr or $\approx 5.34 * 10^{32}$ atoms/yr ^{9}Be globally from chemically weathered crust. Comparing this again to the annual supply of cosmogenic ^{10}Be, we may expect a modern ocean ^{10}Be/^{9}Be ratio less than approximately 1.2×10^{-8} — closer, but still an order of magnitude lower than observed. Consequently, the constancy of the fraction of weathered and eroded ^{9}Be that enters the open ocean is of paramount importance to the accuracy of the seawater ^{10}Be/^{9}Be proxy.

While there is some evidence that the dissolved and reactive proportion of eroded crustal ^{9}Be may be approximately constant in modern fluvial settings (*99*), the proportion of this ^{9}Be that reaches the open ocean has recently become the subject of some debate (*28*)—and the mobility of eroded ^{9}Be in glacial context and over long timescales is even less well understood. In

general, the aqueous mobility of Be is highly dependent on pH (*96*), with an upper limit under many conditions set by the solubility of $BeOH_2$, for which $\log_{10} K_{sp} \approx -21.16$ (*100*). Correspondingly, observed dissolved Be concentrations in terrestrial surface waters, as seen in Fig. 5, decline by about three to five orders of magnitude between pH 4 and pH 8, or up to a factor of 2 per 0.15 pH units at the theoretical solubility limit. Consequently, over long timescales, increasing seawater pH as a result of increasing silicate weathering rate may be expected to counteract increasing ^{9}Be supply, obscuring the relationship between ocean ^{10}Be/^{9}Be ratios and silicate weathering rate.

Over shorter timescales, the conditions of weathering prior to sediment reaching the ocean may be important as well. In nonglacial erosion, exposed crust is generally first weathered at the surface prior to removal by physical erosion. In comparison to the ocean, with a preindustrial average pH around 8.2 (*51*), rainwater (pH ∼4.77 (*50*)) and soil pore water (highly variable, but with a median of ∼ pH 5.72 (*50*)) are on average significantly more acidic – and in the case of soils, are enriched in humic and fulvic acids which likely further promote Be mobility (*52*). In contrast, glacially eroded crust is generally finely comminuted but relatively unweathered. While glacially eroded silicates supplied to the ocean may subsequently undergo chemical weathering in suspension due to their finely comminuted form, any such weathering will occur at ocean pH conditions, likely diminishing the fraction of eroded ^{9}Be that partitions into the aqueous phase and thus masking the impact of glacial erosion on seawater ^{10}Be/^{9}Be.

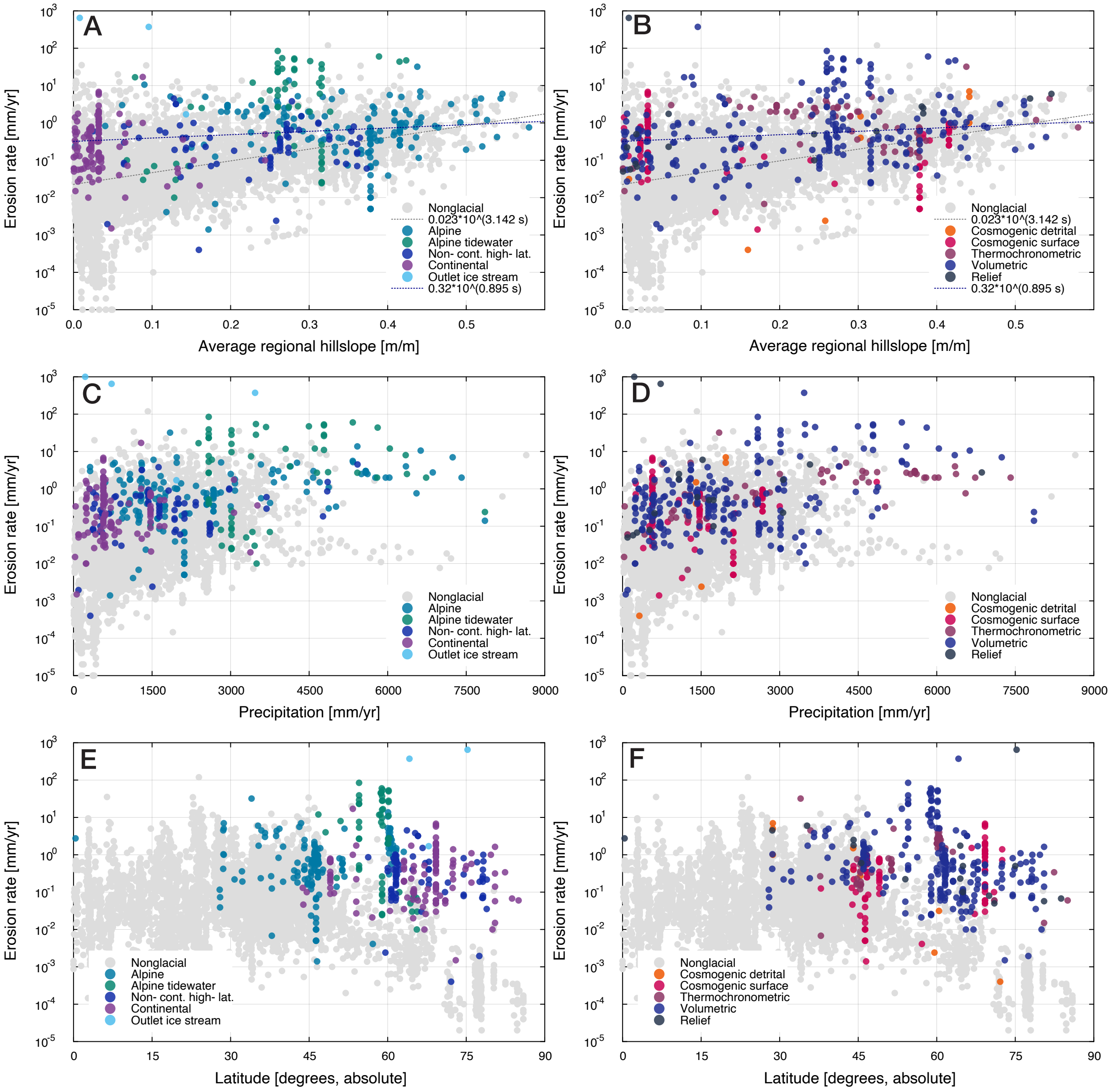

Figure S1: Erosion rate as a function of (A-B) average regional hillslope, (C-D) precipitation and (E-F) latitude. Glacial erosion rates are colored alternately by type (left) or by method (right).

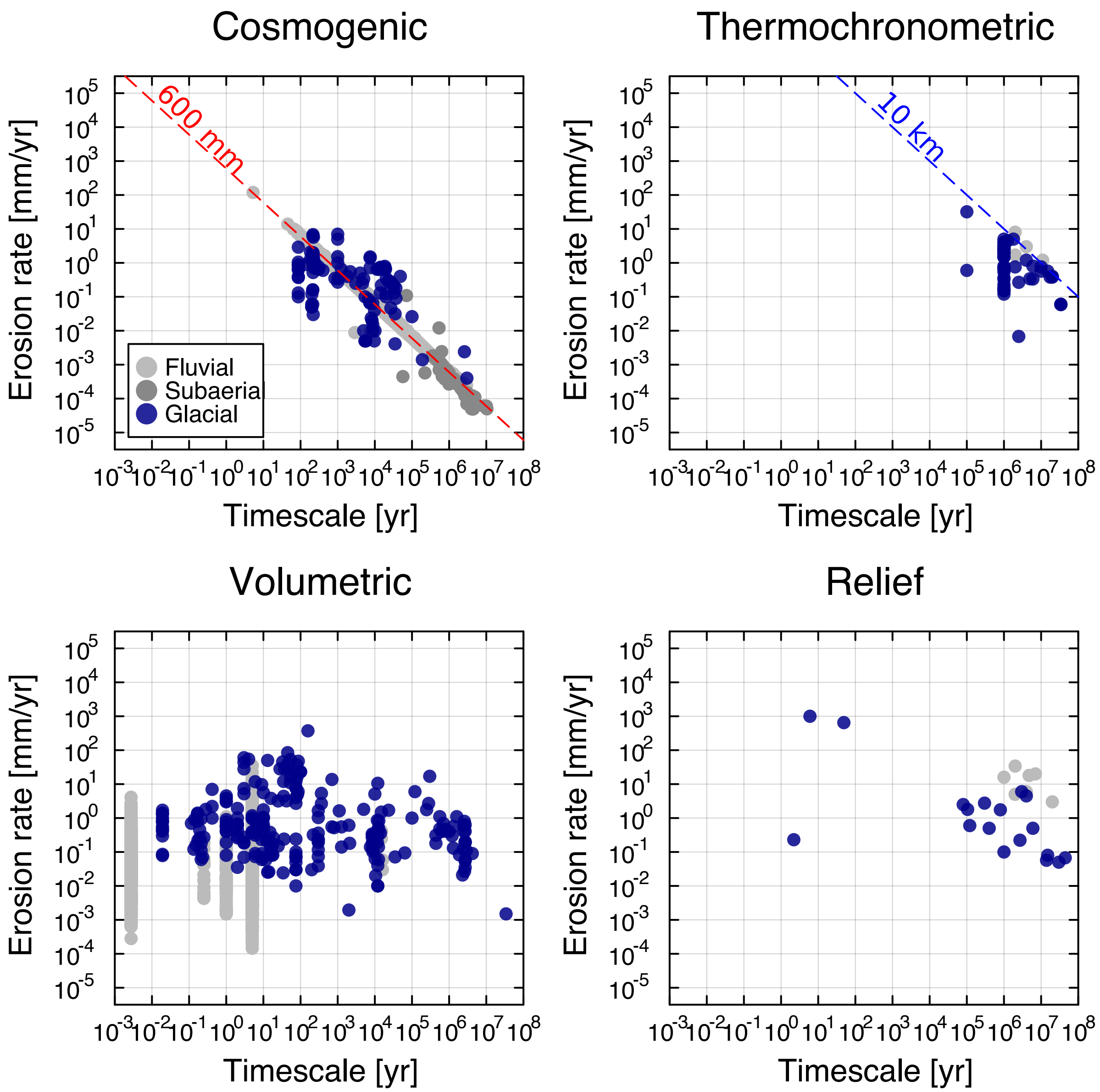

Figure S2: Erosion rate versus timescale, showing timescale dependence, by measurement method.

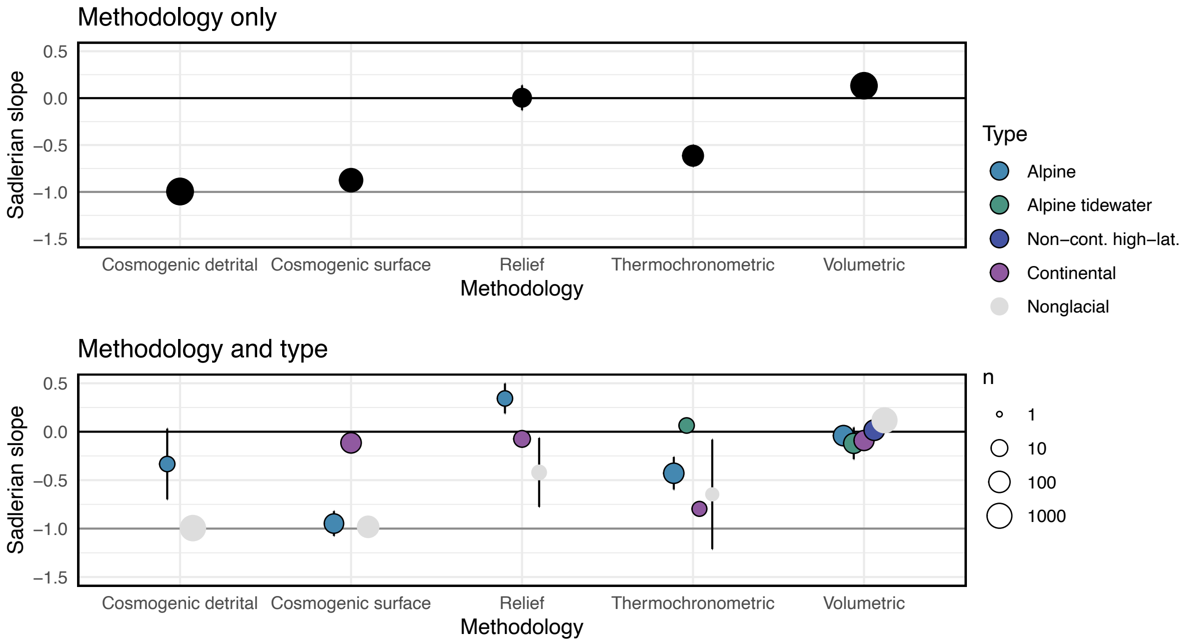

Figure S3: Sadlerian slopes delineated by methodology (upper panel) and by both methodology and glacier type (bottom panel). Marker size is scaled by the number of samples in that category. Where visible, error bars representing the standard error are shown.

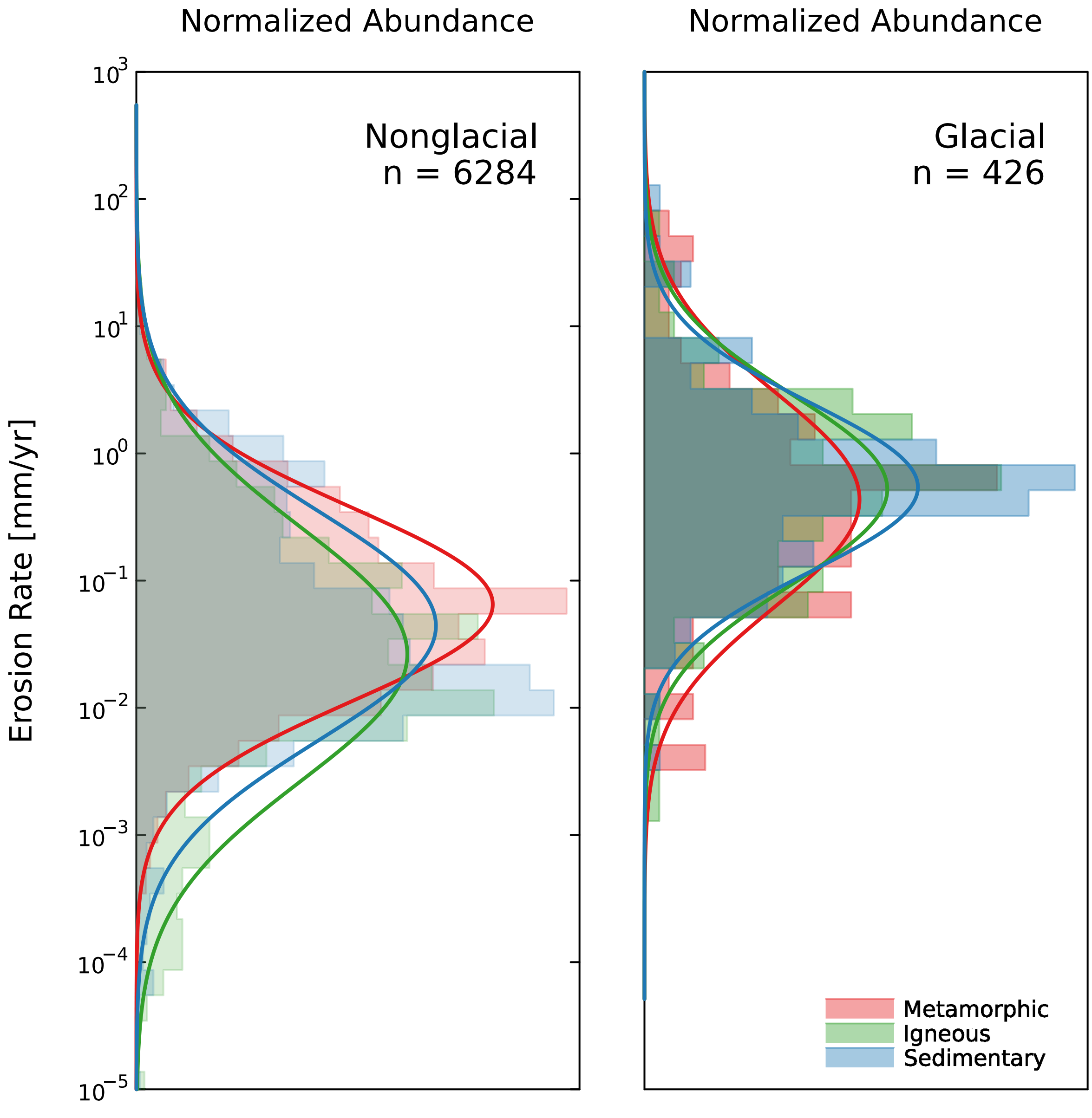

Figure S4: Distribution of glacial and nonglacial erosion rates by bedrock lithology. Glacial erosion is faster than nonglacial erosion across all lithologies.

Table S1: Pairwise ANCOVA results of log-transformed erosion rates by methodology. Bold p-values indicate statistical significance (p-value<0.05).

**Methodology 1**	**Methodology 2**	**Mean difference (glacial only)**	**p-value (glacial Only)**	**Mean difference (glacial + nonglacial)**	**p-value (glacial + nonglacial)**
Cosmogenic detrital	Cosmogenic surface	-0.5984	**0.0422**	-0.1208	**0.0006**
Cosmogenic detrital	Relief	-0.5706	0.0625	-0.9995	**0.0000**
Cosmogenic detrital	Thermochronometric	-0.2889	0.8027	-0.3813	**0.0111**
Cosmogenic detrital	Volumetric	-0.8676	**0.0001**	-1.1276	**0.0000**
Cosmogenic surface	Relief	0.0278	0.9994	-0.8787	**0.0000**
Cosmogenic surface	Thermochronometric	0.3095	0.4819	-0.2605	0.1961
Cosmogenic surface	Volumetric	-0.2693	**0.0179**	-1.0068	**0.0000**
Relief	Thermochronometric	0.2816	0.5834	0.6183	**0.0001**
Relief	Volumetric	-0.2971	**0.0089**	-0.1281	0.5091
Thermochronometric	Volumetric	-0.5787	**0.0070**	-0.7464	**0.0000**

Data S1: Glacial Erosion Rates. A spreadsheet of all published glacial erosion rates compiled for this study, along with information about where they were collected from (region, name of glacier, elevation, latitude, and longitude), environmental conditions (precipitation, slope, type of glacier, and lithology, where available), the methodology used to calculate them, the area and time interval over which they were calculated, and additional information on any sediment fluxes or exhumation rates the authors calculated. We also include tabs with information about how we classified studies into different regions, assigned them different lithologies, and a tab compiling which studies have specific bearing on Snowball Earth.

Data S2: Fluvial and Subaerial Erosion Rates. A spreadsheet of all published fluvial and subaerial erosion rates compiled for this study, along with information about where they were collected from (region, elevation, latitude, and longitude), environmental conditions (precipitation, slope, and lithology, where available), the methodology used to calculate them, and the area and time interval over which they were calculated. More information about the previously published compilations of fluvial erosion rates we incorporate into this study is also available in subsequent tabs.
